# ﻿Geometric morphometry of the *Rhodniusprolixus* complex (Hemiptera, Triatominae): patterns of intraspecific and interspecific allometry and their taxonomic implications

**DOI:** 10.3897/zookeys.1202.108157

**Published:** 2024-05-23

**Authors:** Ana Carolina P. C. Alvarez, Carolina Dale, Cleber Galvão

**Affiliations:** 1 Laboratório de Entomologia, Instituto Oswaldo Cruz, FIOCRUZ, Av. Brasil 4365, Pavilhão Mourisco, sala 214, Rio de Janeiro, RJ, 21040-360, Brazil Instituto Oswaldo Cruz Rio de Janeiro Brazil; 2 Laboratório Nacional e Internacional de Referência em Taxonomia de Triatomíneos, Instituto Oswaldo Cruz, FIOCRUZ, Av. Brasil 4365, Pavilhão Rocha Lima, sala 507, Rio de Janeiro, RJ, 21040-360, Brazil Instituto Oswaldo Cruz Rio de Janeiro Brazil

**Keywords:** Chagas disease, entomological collections, *Rhodniusnasutus*, *R.neivai* complex, taxonomy, vector

## Abstract

In the subfamily Triatominae, the genus *Rhodnius* is one of the most studied, not only because of its epidemiological importance, but also because of the difficulty in differentiating its species. Currently, one of the strategies to control Chagas disease, besides other initiatives such as the analysis of donated blood, is focused on fighting the vector. Correctly identifying triatomines is essential for the entomoepidemiological surveillance of Chagas disease. The objective of the present work was to compare the species of the *R.prolixus* complex using geometric morphometry of hemelytra and heads to evaluate the patterns of intraspecific and interspecific allometry and their taxonomic implications. This method can help in the diagnosis of close species, whose morphological characteristics are insufficient for correct identification. Specimens from five different collections were used, covering the species included in the *R.prolixus* complex (*R.barretti*, *R.dalessandroi*, *R.domesticus*, *R.marabaensis*, *R.milesi*, *R.montenegrensis*, *R.nasutus*, *R.neglectus*, *R.neivai*, *R.prolixus* and *R.robustus*). Morphometric analyses indicated that the hemelytra are not structures with good resolution for separating species and, for this reason, the use of the heads proved to be more adequate for this group (thus allowing differentiation of all species of the *R.prolixus* complex). The results suggest that *R.milesi* is a variant of *R.neglectus* and confirms that *R.prolixus* and *R.robustus* are distinct species. Furthermore, we propose the creation of the *R.neivai* complex comprising *R.domesticus* and *R.neivai*.

## ﻿Introduction

Chagas disease is endemic to, and one of the most serious diseases in Latin America, with the number of cases still underreported (Dumonteil and Herrera 2017; Nascimento et al. 2019). Despite having several forms of transmission, including an increase in cases related to oral transmission, the classical form of transmission is through the infected excrements of insect vectors of the subfamily Triatominae (Hemiptera: Reduviidae) infected with the parasite *Trypanosomacruzi* (Chagas, 1909) (Kinetoplastida, Trypanosomatidae) (Dias and Schofield 1999; Schmunis 1999; de Fuentes-Vicente et al. 2023). Triatominae currently includes five tribes, 18 genera and 160 species (Poinar 2019; Alevi et al. 2020; Téllez-Rendón et al. 2023; Zhao et al. 2023), among which the tribes Triatomini and Rhodniini have major epidemiological relevance (Lent and Wygodzinsky 1979; Galvão et al. 2003; Vallejo et al. 2009).

Rhodniini is composed of the genera *Rhodnius* Stål, 1859 and *Psammolestes* Bergroth, 1911. *Rhodnius* is one of the best studied genera, not only for its epidemiological significance, but also for the difficultly in distinguishing its species and/or defining species limits (Lent and Jurberg 1969; Lent and Wygodzinsky 1979; Coutinho 2013). It is well characterized by the insertion of its antennae on the distal portion of the head and by the presence of post-ocular callosities, but its species are difficult to differentiate morphologically although they are genetically distinct (Neiva and Pinto 1923; Lent 1948; Pavan and Monteiro 2007; Coutinho 2013; Zhao et al. 2021). Despite the existence of identification keys, e.g., Lent and Wygodzinsky (1979) and Galvão and Dale (2014), the differentiation of these species is still a major obstacle. At present, there are 21 species considered as valid that are grouped into three complexes following molecular phylogenies based on different sequences (16S mitochondrial rDNA, cytochrome b (Cytb) and 28S nuclear rRNA): *R.pallescens*, *R.pictipes* and *R.prolixus* (Table 1) (Maia da Silva et al. 2004; Zhao et al. 2021).

**Table 1.** *Rhodnius* species complexes according to Zhao et al. (2021).

Complex	Species
***Rhodniusprolixus***	*Rhodniusbarretti* Abad-Franch, Palomeque & Monteiro, 2013
	*Rhodniusdalessandroi* Carcavallo & Barreto, 1976
	*Rhodniusdomesticus* Neiva & Pinto, 1923
	*Rhodniusmilesi* Carcavallo, Rocha, Galvão & Jurberg, 2001
	*Rhodniusmarabaensis* dos Santos Souza et al., 2016
	*Rhodniusmontenegrensis* da Rosa et al., 2012
	*Rhodniusnasutus* Stål, 1859
	*Rhodniusneglectus* Lent, 1954
	*Rhodniusneivai* Lent, 1953
	*Rhodniusprolixus* Stål, 1859
	*Rhodniusrobustus* Larrousse, 1927
***Rhodniuspictipes***	*Rhodniusamazonicus* Almeida, Santos & Sposina, 1973
	*Rhodniusbrethesi* Matta, 1919
	*Rhodniusmicki* Zhao, Galvão & Cai, 2021
	*Rhodniusparaensis* Sherlock, Guitton & Miles, 1977
	*Rhodniuspictipes* Stål, 1872
	*Rhodniusstali* Lent, Jurberg & Galvão, 1993
	*Rhodniuszeledoni* Jurberg, Rocha & Galvão, 2009
***Rhodniuspallescens***	*Rhodniuscolombiensis* Mejía, Galvão & Jurberg, 1999
	*Rhodniusecuadoriensis* Lent & León, 1958
	*Rhodniuspallescens* Barber, 1932

The *Rhodniusprolixus* complex was, initially, erected by Carcavallo et al. (2000) with the species *R.domesticus*, *R.nasutus*, *R.neglectus*, *R.prolixus* and *R.robustus*. Zhao et al. (2021) updated the complexes using molecular data, geographical distribution patterns and morphometric analyses. As a result, the number of species belonging to the *R.prolixus* complex was increased from five to eleven, adding to the previous species: *R.barretti*, *R.dalessandroi*, *R.marabaensis*, *R.milesi*, *R.montenegrensis* and *R.neivai*.

Currently, one of the strategies to control the disease, besides other initiatives such as the analysis of donated blood, is focused on fighting the vector, making the correct identification of triatomines essential for the entomoepidemiological surveillance of Chagas disease (The Pan American Health Organization 2023). Studies demonstrate the importance of using other techniques for the identification of species, especially close ones, in triatominae taxa (Gurgel-Gonçalves et al. 2011; Depickère et al. 2020). Over the years, research using new approaches to species differentiation have been published, such as the study of genitalia (e.g., Lent and Jurberg 1969), analysis of the exochorion of eggs (e.g., Barata 1981), description of nymphs (e.g., Rocha et al. 2005), scanning electron microscope (e.g., da Rosa et al. 2014), morphometry (e.g., Gurgel-Gonçalves et al. 2008), isoenzyme analyses (e.g., Dujardin et al. 1999), cytogenetics (e.g., Ravazi et al. 2021), matrix-assisted laser desorption/ionization-time of flight mass spectrometry (MALDI-TOF MS) (Souza 2020), transcriptome (de Carvalho et al. 2017), and more recently DNA analyses (e.g., Montiel et al. 2021). However, even with the advancement of these tools, some species still do not show enough characters for easy diagnosis (Monteiro et al. 2000; Fornel and Cordeiro-Estrela 2012; Coutinho 2013).

The objective of the present work is to compare the species of the *R.prolixus* complex using geometric morphometry of hemelytra and heads to evaluate the patterns of intraspecific and interspecific allometry and their taxonomic implications.

## ﻿Material and methods

### ﻿Species samples

The specimens used in the study are from five distinct entomological collections:

Coleção de Triatomíneos do Instituto Oswaldo Cruz (**CTIOC**), of the Laboratório Nacional e Internacional de Referência em Taxonomia de Triatomíneos (**LNIRTT**), FIOCRUZ, Rio de Janeiro, Brazil;

Coleção Entomológica do Instituto Oswaldo Cruz (**CEIOC**), Laboratório de Entomologia (**LABE**), FIOCRUZ, Rio de Janeiro, Brazil;

Entomological collection of the Museum für Naturkunde – Leibniz Institute for Evolution and Biodiversity Science, Berlin, Germany;

Coleção de Triatominae Dr. José Maria Soares Barata, Faculdade de Ciências Farmacêuticas, UNESP, Araraquara, São Paulo, Brazil;

Coleção do Centro de Pesquisa René Rachou (**CPqRR**), FIOCRUZ, Minas Gerais, Brazil. (Suppl. material 1).

For taxonomic identification of adults, the dichotomous keys from Galvão and Dale (2014) were used.

### ﻿Image acquisition and data analysis

**Figure 1.**
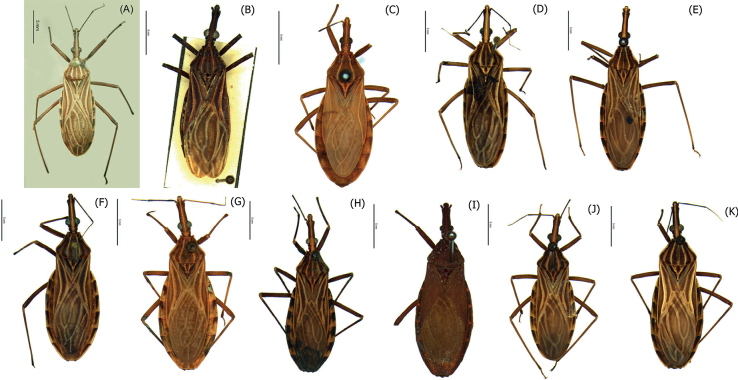
Habitus, dorsal view of **A***Rhodniusbarretti***B***Rhodniusdalessandroi***C***Rhodniusdomesticus***D***Rhodniusmarabaensis***E***Rhodniusmilesi***F***Rhodniusmontenegrensis***G***Rhodniusnasutus***H***Rhodniusneglectus***I***Rhodniusneivai***J***Rhodniusprolixus***K***Rhodniusrobustus*. Scale bars: 5 mm.

The hemelytra and the heads of specimens belonging to 11 valid species belonging to the *Rhodniusprolixus* complex were photographed using the Leica Automounting Magnifier (DMC 2900): *R.barretti*, *R.dalessandroi*, *R.domesticus*, *R.marabaensis*, *R.milesi*, *R.montenegrensis*, *R.nasutus*, *R.neglectus*, *R.neivai*, *R.prolixus* and *R.robustus* (Fig. 1).

### ﻿Geometric morphometric analysis

**Figure 2.**
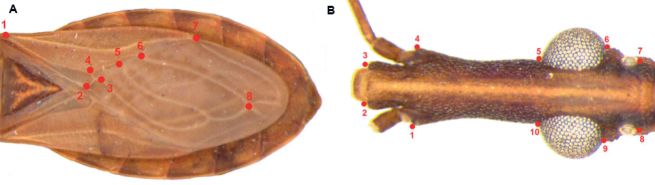
Landmarks in **A** hemelytra of *R.domesticus* and **B** head of *R.prolixus*.

In this study, the geometric morphometry method was employed. The technique involved utilizing previously acquired images and the TPSdig software ver. 2.31 (Rohlf 2005). Following the method of Dujardin (2019), eight type I landmarks were selected on the hemelytra (except for *R.barretti* due to the lack of specimens in the collections) and ten on the heads of each specimen of the *R.prolixus* complex (Fig. 2A, B) (Suppl. material 2). According to Dujardin (2019), type I landmarks “may be considered as anatomical points or patches recognizable from one individual to another”. All landmarks used were identified as regions where structural features converge.

### ﻿Data transformation and analysis

Using TPSrelw software ver. 1.75 (Rohlf 2005), the data were transformed into numerical coordinates and stored as weighted matrix in an NTS format. Once the matrix was generated, the centroid size, as well as the X and Y uniform components, were calculated for each specimen, following the method outlined by (Coutinho 2017). The X and Y coordinate landmarks underwent Procrustes superimposition (Bookstein 1991), followed by Thin Plate Spline analysis, and subsequently a discriminant analysis. Multivariate analyses and factorial maps were constructed using JMP software ver. 17 (Institute 2000).

### ﻿Multivariate analysis and software

The data underwent a multivariate principal component analysis (PCA) to show the variability of shapes within the genus, constructing a factor map. Subsequently, the covariance matrix generated by Procrustes coordinate analysis and a multivariate analysis of variance (MANOVA) were conducted to assess shape variation. Species relationships were determined by canonical components (CVA), which can be useful to find shape features and to distinguish the groups of species included in the complex. The statistical tests (including Wilks’ Lambda, Pillai’s Trace, Hotelling-Lawley and Roy’s Max Root) for both hemelytra and head analyses were automatically performed using JMP. Using the same software, factorial maps of principal and canonical components were generated, along with dendrograms employing Mahalanobis distances for cluster analysis of both structures.

## ﻿Results

### ﻿Geometric morphometry of hemelytra

Wilks’ lambda test for analysis of hemelytra size variation revealed significant differences (*p* < 0.0001) among species (Table 2). PCA resulted in the sum of the values of the first (PC1) and second (PC2) principal components equivalent to 68% of the total shape variability (PC1 = 53.80% and PC2 = 14.20%).

**Table 2.** Statistical tests performed by JMP.

Test	Value	Approx. F	NumDF	DenDF	Prob>F
**Wilks’ Lambda**	0.2901718	5.4757	36	503.9	<.0001
**Pillai’s Trace**	0.9645573	4.8371	36	548	<.0001
**Hotelling-Lawley**	1.6451934	6.0668	36	352.37	<.0001
**Roy’s Max Root**	0.977472	14.8793	9	137	<.0001

**Figure 3.**
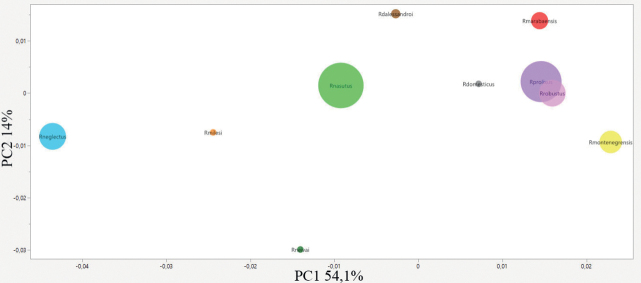
Factorial map containing the principal components of the hemelytra where each species is represented by circles. Brown – *Rhodniusdalessandroi*; Gray – *Rhodniusdomesticus*; red – *Rhodniusmarabaensis*; orange – *Rhodniusmilesi*; yellow – *Rhodniusmontenegrensis*; light green – *Rhodniusnasutus*; light blue – *Rhodniusneglectus*; dark green – *Rhodniusneivai*; lilac – *Rhodniusprolixus*; light pink – *Rhodniusrobustus* (Suppl. material 2)

**Figure 4.**
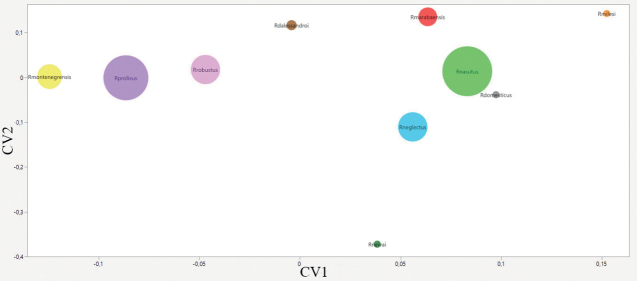
Factorial map containing the canonical variation of the hemelytra where each species is represented by circles. Brown – *Rhodniusdalessandroi*; gray – *Rhodniusdomesticus*; red – *Rhodniusmarabaensis*; orange – *Rhodniusmilesi*; yellow – *Rhodniusmontenegrensis*; light green – *Rhodniusnasutus*; light blue – *Rhodniusneglectus*; dark green – *Rhodniusneivai*; lilac – *Rhodniusprolixus*; light pink – *Rhodniusrobustus* (Suppl. material 2).

In the plots it is possible to observe the distant distribution of each species. We can see this kind of distribution on the PCA map (Fig. 3) where only *R.robustus* and *R.prolixus* are overlapping. On the CVA map (Fig. 4), there is no species overlap.

**Figure 5.**
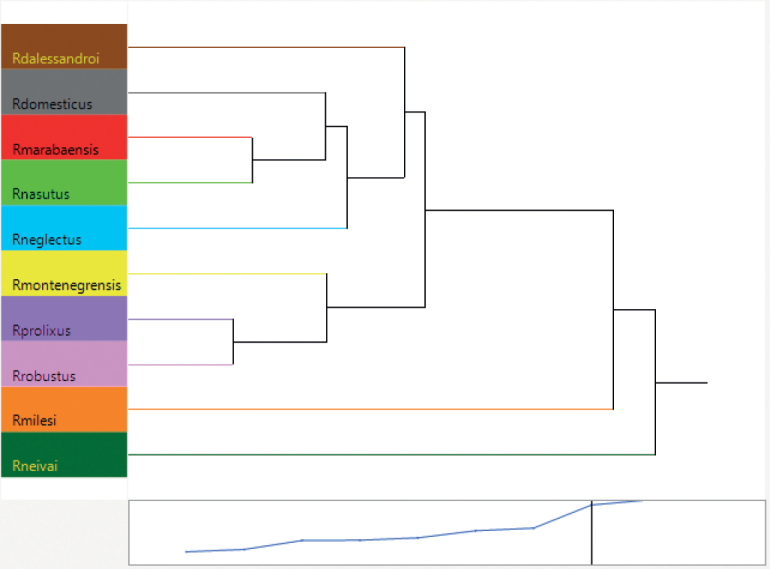
Dendogram produced by hemelytra cluster analysis of the species of the *Rhodniusprolixus* complex, except *Rhodniusbarretti* (Suppl. material 2).

The cluster analysis, using the mean distances between species (Mahalanobis distances), produced a dendogram (Fig. 5) that formed a group including *R.nasutus*, *R.marabaensis* and *R.domesticus*, connected to *R.neglectus* and *R.dalessandroi*; a group formed by *R.prolixus* and *R.robustus* connected to *R.montenegrensis*; *Rhodniusmilesi* and *R.neivai* as outgroup species.

### ﻿Head geometric morphometry

Wilk’s Lambda test for head size variation analysis revealed significant differences (p < 0.0001) among species (Table 3). The PCA showed as a result the sum of CP1 and CP2 values equivalent to 77% of the total shape variability (CP1 = 63.50% and CP2 = 13.50%).

**Table 3.** Statistical tests performed by JMP.

Test	Value	Approx. F	NumDF	DenDF	Prob>F
**Wilks’ Lambda**	0.0180787	35.0464	40	745.06	<.0001
**Pillai’s Trace**	2.0449084	20.8142	40	796	<.0001
**Hotelling-Lawley**	10.710766	52.1415	40	536.68	<.0001
**Roy’s Max Root**	6.5944871	131.2303	10	199	<.0001

**Figure 6.**
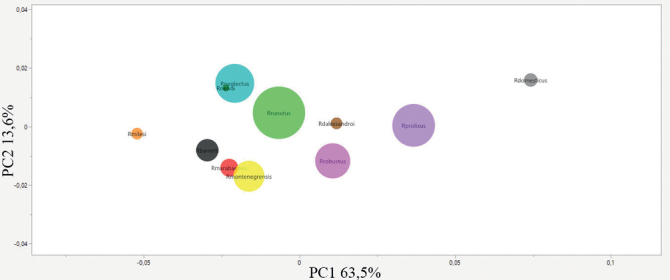
Factorial map containing the principal components of the head where each species is represented by circles. Brown – *Rhodniusdalessandroi*; gray – *Rhodniusdomesticus*; red – *Rhodniusmarabaensis*; orange – *Rhodniusmilesi*; yellow – *Rhodniusmontenegrensis*; light green – *Rhodniusnasutus*; light blue – *Rhodniusneglectus*; dark green – *Rhodniusneivai*; lilac – *Rhodniusprolixus*; light pink – *Rhodniusrobustus* (Suppl. material 2).

**Figure 7.**
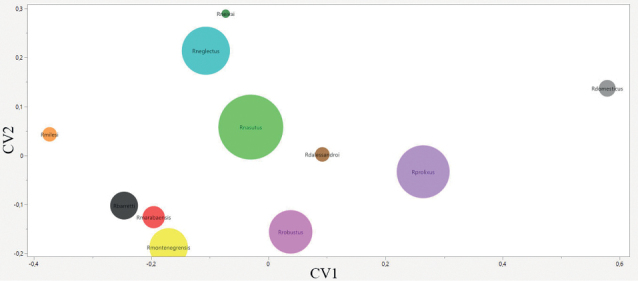
Factorial map containing the canonical components of the head where each species is represented by circles. Brown – *Rhodniusdalessandroi*; gray – *Rhodniusdomesticus*; red – *Rhodniusmarabaensis*; orange – *Rhodniusmilesi*; yellow – *Rhodniusmontenegrensis*; light green – *Rhodniusnasutus*; light blue – *Rhodniusneglectus*; dark green – *Rhodniusneivai*; lilac – *Rhodniusprolixus*; light pink – *Rhodniusrobustus* (Suppl. material 2).

The positioning of each species is observed in the factorial maps of the PCA (Fig. 6) where we see a clear overlap between *R.neglectus* and *R.neivai*; *Rhodniusprolixus* appears distant from *R.robustus* and the group formed by *R.marabaensis*, *R.montenegrensis* and *R.barretti*; *Rhodniusdomesticus* appears as an outgroup species. On the CVA map (Fig. 7) we again see *R.prolixus* distant from *R.robustus* and the group formed by *R.barretti*, *R.marabaensis* and *R.montenegrensis*; *Rhodniusdomesticus* appears again as an outgroup species.

**Figure 8.**
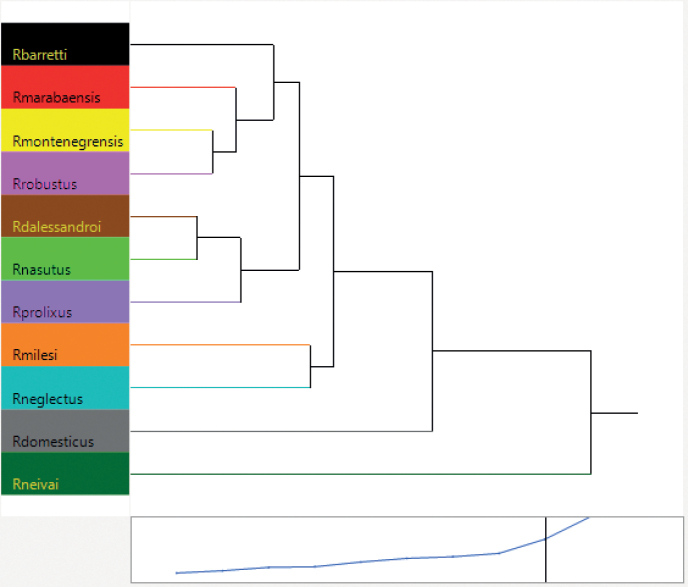
Dendogram produced by head cluster analysis of the species of the *Rhodniusprolixus* complex (Suppl. material 2).

The cluster analysis using the mean distances among species generated a dendogram (Fig. 8) that reinforces *R.domesticus* and *R.neivai* as outer groups. In addition, we can visualize a group formed by two inner groups, the first being a group including *R.barretti*, *R.marabaensis*, *R.montenegrensis* and *R.robustus* and the second inserting *R.dalessandroi* close to *R.nasutus* and *R.prolixus*; *Rhodniusmilesi* is directly linked to *R.neglectus*.

## ﻿Discussion

Despite advances in studies related to the taxonomy and systematics of Triatominae, some species still lack sufficient characters for easy diagnosis, such as those belonging to the genus *Rhodnius* (Monteiro et al. 2000; Fornel and Cordeiro-Estrela 2012; Coutinho 2013). Although this genus is easily identified using morphological characters, the differentiation of species is still a major challenge (Neiva and Pinto 1923; Lent 1948; Galvão 2014). Due to their similarities, Zhao et al. (2021) grouped the species of this genus into three complexes according to their distribution: *R.pallescens*, *R.pictipes* and *R.prolixus*. The *R.pallescens* group is considered as trans-Andean, found on the western side of the Andes, while the *R.pictipes* is cis-Andean, distributed on the eastern side of the Andes and also in the Amazon region. In the *R.prolixus* complex, ten species (*R.barretti*, *R.dalessandroi*, *R.domesticus*, *R.marabaensis*, *R.milesi*, *R.montenegrensis*, *R.nasutus*, *R.neglectus*, *R.prolixus* and *R.robustus*) are distributed in the same cis-Andean region of the *R.pictipes* group, and only *R.neivai* has trans-Andean populations (Filée et al. 2022).

Some species in the *R.prolixus* complex are difficult to differentiate using only morphological characters, which can lead to taxonomic conflicts (Filée et al. 2022). Gurgel-Gonçalves et al. (2011) found in *R.neglectus* variations comparing sylvatic and laboratory colonies. Some variations (e.g., size or chromatic) can lead to misidentification, and occasionally to under-reporting of Chagas’ disease transmission cases related to different species (Dias 2007; Dias et al. 2014). In cases like those, when chromatic and size variations are significant, geometric morphometry could be a useful method to differentiate species, as seen in Soto Vivas et al. (2007), Gurgel-Gonçalves et al. (2011), Abad-Franch et al. (2021) and Cruz et al. (2023). In addition, geometric morphometry is an important method in studies using specimens from entomological collections, in which molecular analysis can sometimes be inefficient, as presented in Dale et al. (2013).

Lent and Wygodzinsky (1979), cited *R.nasutus* and *R.neglectus* as examples of difficult differentiation, because their phenotypical similarities. The results of head geometric morphometry shows a group formed by *R.nasutus*, *R.dalessandroi* and *R.prolixus*, corroborating the results of Coutinho (2013) who compared the species of *Rhodnius* using the mitochondrial gene cytochrome oxidase I (COI). Meanwhile, *R.neglectus* appears closely related to *R.milesi*, which was described by Valente et al. (2001) as close to *R.dalessandroi*, despite its distant geographical distribution. Coutinho (2013) used COI to verify the great genetic similarity between *R.milesi* and *R.neglectus*, the first being considered as a variant of the second. This result is also found in Monteiro et al. (2018), who used Cytb and ITS-2 sequences. Despite the distribution of the two species in the factorial maps, the relationships between *R.neglectus* and *R.milesi* found in the dendogram generated by the heads, agree with those found by Coutinho (2013) and Monteiro et al. (2018) in grouping these two species together.

The dendogram generated by cluster analysis of the head of *R.dalessandroi*, described by Carcavallo and Barreto (1976) as close to *R.brethesi* (a species of the *R.pictipes* complex), appear in the group next to *R.nasutus* and *R.prolixus*, and next to a group formed by *R.neglectus*, *R.nasutus*, *R.marabaensis* and *R.domesticus*. *Rhodniusdalessandroi* have little information in the literature, with Lent and Wygodzinsky (1979) citing that “the published description and illustrations of this *Rhodnius* are not sufficient to recognize it.”

The identification key from Lent and Wygodzinsky (1979) and Galvão and Dale (2014), using external morphology, places *R.robustus* and *R.prolixus* as morphologically similar species (which makes their identification difficult). Our analyses of geometric morphometry of the heads showed that these two species are distinct. In fact, *R.robustus* is close to *R.montenegrensis* and *R.prolixus* close to *R.nasutus* and *R.dalessandroi*. Although, in contrast Schofield and Dujardin (1999) considered *R.prolixus* a species domiciliary adapted from a wild *R.robustus* lineage; the results found in the present work agrees with Monteiro et al. (2003), who reiterated that both species were independent taxa (analyzing Cytb), and with Feliciangeli et al. (2007), who comparing the two species using geometric morphometry of hemelytra, showed a possible wild origin of *R.prolixus*.

Monteiro et al. (2003) considered *R.robustus* a paraphyletic group and formed by at least four cryptic species (represented by the author as lineages I, II, III and IV). De Carvalho et al. (2017) used transcriptomic analysis to demonstrate that *R.montenegrensis* and *R.robustus* represented distinct species. Monteiro et al. (2018) suggested that lineage II of *R.robustus* was described as *R.montenegrensis* and lineage III as *R.marabaensis*. On the other hand, Brito et al. (2019), as well using transcriptomic analysis, observed that *R.montenegrensis* would be genetically indistinguishable from a variant of *R.robustus* II (specimens from Bolivia, Brazil and Ecuador). Brito et al. (2019) also hypothesized that the colonies used as a reference in the description of *R.montenegrensis* were probably a mixture of colonies of *R.prolixus* and *R.robustus*. In the present study, we verified that, in the geometric morphometry of the heads, *R.montenegrensis* was not as close as expected to *R.robustus* and/or *R.prolixus* in the bubble plot (factorial) map, but directly linked to *R.robustus* and closely related to *R.marabaensis* and *R.barretti* in the dendogram. In the cluster analysis using hemelytra, the species appear external to the group formed by *R.prolixus* and *R.robustus*.

Both hemelytra and head geometric morphometry show *R.neivai* and *R.domesticus* as outgroup species. These results corroborate those obtained by Schofield and Dujardin (1999), Monteiro et al. (2000) and de Paula et al. (2007). These species, considered ancient and isolated, are found geographically far from others present in the *R.prolixus* complex. *Rhodniusdomesticus* is commonly found in bromeliads of the Brazilian Atlantic Forest and the *Rhodniusneivai* is found near the Andes Mountains (in Colombia and Venezuela) and in the Maracaibo basin (Abad-Franch et al. 2009; Pita et al. 2013; Monteiro et al. 2018). This fact may justify why these species have such specific characteristics and are located as outliers in the analyses. Carcavallo et al. (2000) cited that *R.domesticus* had sufficient morphological characters to be considered a separate taxon, while Monteiro et al. (2000) stated that *R.neivai* has no support, based on Cytb sequences, to be associated with the *R.prolixus* complex. Schofield and Dujardin (1999) even proposed that *R.neivai* should be grouped with the *R.pictipes* complex. Thus, according to the evidence found in the present work and corroborating the results of Schofield and Dujardin (1999), Monteiro et al. (2000) and de Paula et al. (2007), we suggest the removal of both species from the *R.prolixus* complex and the establishment of a *R.neivai* complex comprising both, *R.domesticus* and *R.neivai*.

Comparing the graphical analysis of the hemelytra and recent published papers as Zhao et al. (2021), we observed that the results using this structure do not always present an adequate resolution for separating the species as expected reflecting molecular phylogeny, indicating that possibly this structure has a lot of homoplasy and very similar morphologies. For this reason, the use of heads to elucidate the differences seems to be more appropriate for this group of species.

## ﻿Conclusion

The use of different taxonomic methods (integrative taxonomy) is increasingly important in taxonomic studies, especially when dealing with closely related species (Alevi et al. 2020). Through geometric morphometry, it was possible to define the morphometric profiles of the species belonging to the *R.prolixus* complex using both structures, hemelytra, and heads (except for *R.barretti*, for which it was not possible to analyze the hemelytra). Using this method, focusing on the heads, it was possible to differentiate all the species used, which include *R.barretti*, *R.dalessandroi*, *R.domesticus*, *R.marabaensis*, *R.milesi*, *R.montenegrensis*, *R.nasutus*, *R.neglectus*, *R.neivai*, *R.prolixus*, and *R.robustus*. These results suggest that *R.milesi* is indeed a variant of *R.neglectus*, emphasizing the need for formal synonymization. They also propose the establishment of the *R.neivai* complex (comprising the species *R.domesticus* and *R.neivai*) (Suppl. material 3) and confirm that *R.prolixus* and *R.robustus* are distinct species.

## ﻿Additional information

### Author ORCIDs

Ana Carolina P. C. Alvarez https://orcid.org/0000-0002-5853-8298

Carolina Dale https://orcid.org/0000-0002-9526-9242

Cleber Galvão https://orcid.org/0000-0003-4027-9205

### Data availability

All of the data that support the findings of this study are available in the main text or Supplementary Information.

## Supplementary materials

Supplementary material 1 Collection numbers of the specimens studied Data type xlsx Explanation note List of collection numbers of the specimens studied. https://binary.pensoft.net/file/1054541This dataset is made available under the Open Database License (http://opendatacommons.org/licenses/odbl/1.0/). The Open Database License (ODbL) is a license agreement intended to allow users to freely share, modify, and use this Dataset while maintaining this same freedom for others, provided that the original source and author(s) are credited.Ana Carolina P. C. Alvarez, Carolina Dale, Cleber Galvão

Supplementary material 2 Colors used to identify the species of *R.prolixus* complex Data type xlsx Explanation note Colors and codes used to identify specimens in hemelytra and head analyzes; Numbers of specimens used on each analyzes. https://binary.pensoft.net/file/1054542This dataset is made available under the Open Database License (http://opendatacommons.org/licenses/odbl/1.0/). The Open Database License (ODbL) is a license agreement intended to allow users to freely share, modify, and use this Dataset while maintaining this same freedom for others, provided that the original source and author(s) are credited.Ana Carolina P. C. Alvarez, Carolina Dale, Cleber Galvão

Supplementary material 3 Establishment of the *R.neivai* complex Data type xlsx Explanation note Comprising the species *R.domesticus* and *R.neivai*. https://binary.pensoft.net/file/1054543This dataset is made available under the Open Database License (http://opendatacommons.org/licenses/odbl/1.0/). The Open Database License (ODbL) is a license agreement intended to allow users to freely share, modify, and use this Dataset while maintaining this same freedom for others, provided that the original source and author(s) are credited.Ana Carolina P. C. Alvarez, Carolina Dale, Cleber Galvão
